# Administration of a glypican‐3 peptide increases the infiltration and cytotoxicity of CD8 ^+^ T cells against testicular yolk sac tumor, associated with enhancing the intratumoral cGAS/STING signaling

**DOI:** 10.1002/cam4.6605

**Published:** 2023-11-20

**Authors:** Junfeng Zhao, Le Qin, Guorong He, Tiancheng Xie, Guanghui Hu, Furan Wang, Hongji Zhong, Jianming Zhu, Yunfei Xu

**Affiliations:** ^1^ Department of Urology, Shanghai Tenth People's Hospital School of Medicine in Tongji University Shanghai China; ^2^ Department of Pediatrics Surgery Ningbo Women and Children's Hospital Ningbo China; ^3^ Department of Pediatrics Surgery The Second Affiliated Hospital and Yuying Children's Hospital of Wenzhou Medical University Wenzhou China; ^4^ Department of Urology, Renji Hospital School of Medicine in Shanghai Jiaotong University Shanghai China

**Keywords:** cGAS/STING signaling, glypican‐3, immunotherapy, peptide vaccine, yolk sac tumor

## Abstract

**Background:**

Glypican‐3 (GPC3) is highly expressed in testicular yolk sac tumor (TYST). GPC3 has been evaluated as a cancer vaccine for some types of tumors, but little is known on the effects of GPC3 peptide‐based therapy on TYST. Here, we evaluated the antitumor effect of GPC3_144‐152_ on TYST and its potential mechanisms.

**Methods:**

GPC3_144‐152_‐specific CD8^+^ T cells were induced by vaccine immunization and examined by ELISPOT. The CD8^+^ T cells were purified for testing their cytotoxicity in vitro against TYST cells by CCK‐8 and TUNEL assays and in vivo against tumor growth. The influence of GPC3_144‐152_ loading and/or cGAS silencing on the tumor growth, apoptosis and cGAS/STING signaling was tested by immunohistochemistry, immunofluorescence, flow cytometry, and Western blot.

**Results:**

Vaccination with GPC3_144‐152_ induced tumor‐specific CD8^+^ T cells that secreted high levels of IFN‐γ and granzyme B, and had potent cytotoxicity against TYST in a dose‐dependent manner. Adoptive transfer of CD8^+^ T cells and treatment with GPC3_144‐152_ significantly inhibited the growth of TYST tumors, but less effective for cGAS‐silenced TYST tumors in vivo. Treatment with GPC3_144‐152_ enhanced the infiltration of CD8^+^ T cells into the tumor environment and their cytotoxicity against TYST tumors in vivo by up‐regulating granzyme B and IFN‐β expression, but down‐regulating GPC3 expression in the tumors. Co‐culture of CD8^+^ T cells with TYST in the presence of exogenous GPC3_144‐152_ enhanced peptide‐specific CD8^+^ T‐cell cytotoxicity in vitro, accompanied by enhancing cGAS, γH2AX, TBK1, and IRF3 phosphorylation in TYST cells, but less effective in cGAS‐silenced TYST cells.

**Conclusions:**

These data indicated that GPC3 peptide‐specific CD8^+^ T cells had potent antitumor activity against TYST tumor, particularly for combined treatment with the peptide, which was partially dependent on the intratumoral cGAS/STNG signaling. GPC3 peptide vaccine may be valuable for the combination treatment of TYST.

## 1 INTRODUCTION

Germ cell tumors (GCTs) are a group of heterogeneous neoplasms with varying degrees of malignancies, although their origin is usually different between pediatric and adult/adolescence.^1^, ^2^ Surgical resection of GCT remains the mainstream treatment, and when combination of platinum‐based chemotherapy and radiotherapy for GCTs seems to be highly curable, these therapeutic strategies usually fail to treat approximately 15%–20% of GCT patients with local relapse and distant metastasis.^3^, ^4^ Moreover, the side effects of radiotherapy and/or chemotherapy on children, such as neurotoxicity, ear and kidney toxicity, seriously affect their quality of life. Therefore, it is urgently needed to develop novel therapeutic strategies for intervention of GCTs.

Immunotherapy may be a potentially attractive option for those with late stage of GCTs. The identification of antigens expressed preferably on tumor cells has enabled us to design specific immunotherapies for malignant tumors by targeting those tumor antigens. Many tumor antigens, such as Wilms tumor antigen and glycoprotein non‐metastatic melanoma protein B, represent potential antigens for the development of peptide vaccines.^5^, ^6^ Additionally, vaccination with tumor antigen peptides for immunotherapy has shown to induce peptide‐specific T‐cell immunity against tumors and has some degrees of antitumor efficacy in clinical trials.^7^, ^8^

Oncofetal antigen glypican‐3 (GPC3) is a member of the glypican family of heparan sulfate proteoglycans and able to attach to the cell surface via the glycosylphosphatidylinositol anchor.^9^ GPC3 is physiologically expressed in various fetal tissues (liver, lung, kidney, and placenta), but its expression is limited in postnatal non‐tumor tissues due to DNA methylation induced epigenetic silencing.^8^ Functionally, GPC3 can regulate cell proliferation, apoptosis and differentiation by modulating various growth factors and signal pathways.^10^ Interestingly, GPC3 is highly expressed in hepatocellular carcinoma (HCC), melanoma, ovarian clear cell carcinoma (OCCC), hepatoblastoma and some types of GCTs with unique histological patterns; GPC3 is diffusely expressed in yolk sac tumors (YSTs), but only focally/moderately expressed in teratoma and isolated syncytiotrophoblasts.^6^, ^8^, ^9^, ^11^, ^12^, ^13^ Besides, GPC3 expression is a biomarker for diagnosis and classification of testicular germ cell tumors (TGCTs). Moreover, GPC3‐bassed vaccines have been testing in different phases of several clinical trials for treatment of HCC,^14^, ^15^ OCCC,^16^ and refractory pediatric solid tumors.^6^ It is notable that YST is the commonest GCT in preadolescent testicular tumors, and expresses high levels of GPC3.^17^ Unfortunately, treatment of TYST still depends on surgical resection and/or chemotherapy.^18^ Currently, there are few studies to explore the potential of GPC3 peptide‐based vaccines for treatment of TYST.

Previous studies have shown that vaccination with a GPC3 peptide can induce antigen‐specific CD4^+^ T and CD8^+^ T‐cell (also known cytotoxic T lymphocyte, CTL) responses in vivo, which are responsible for antitumor effects.^19^, ^20^, ^21^, ^22^ Furthermore, increasing evidence indicates that the cGAS‐cGAMP‐STING pathway responds to DNA damage and mediates inflammation and antitumor immunity by recruiting CTLs into the tumor microenvironment.^23^, ^24^, ^25^

In this study, we tested the hypothesis that vaccination with a GPC3 peptide (GPC3_144‐152_) might activate the cGAS/STING signaling in TYST cells, and enhance the antitumor immune responses of GPC3‐specific cytotoxic T cells (CTLs). We found that GPC3_144‐152_ had potent immunogenicity and induced T‐cell responses to TYST tumor in vitro and in vivo. Moreover, GPC3_144‐152_‐induced CTL recruitment and infiltration into the tumor microenvironment were dependent on activation of the intratumoral cGAS/STING pathway.

## 2 MATERIALS AND METHODS

### 2.1 Mice

Male BALB/c (nu/nu) mice at 4 weeks old were obtained from Shanghai Slaccas. Male B‐HLA‐A2.1 mice (C57BL/6‐B2m^*tm2(B2M/HLA‐A2.1/H2‐D)*^) in 6–7 weeks of age were obtained commercially from Biocytogen. All mice were raised in a specific pathogen‐free condition and acclimated for 1–2 weeks before the experiment. The animal experiments were conducted, according to the Animal Care and Research guidelines of the National Institutes of Health (NIH) and approved by the Animal Care Ethical Committee of our Hospital (2020‐ky‐023).

### 2.2 Cell lines

Human TYST cell line was generated from a pediatric patient with TYST,^18^ and TYST cells at passage 79 were a gift provided by Prof. Xiaoming Chen in the Pediatric Surgery Laboratory of the Second Affiliated Hospital of Wenzhou Medical University. Human colon cancer SW620 (GPC3^−^, as control cells) cells were from Cell Bank of Chinese Academy of Sciences. TYST and SW620 cells were cultured in RPMI 1640 (GIBCO) and L‐15 medium supplemented with 10% fetal bovine serum (FBS), 100 U/mL penicillin, and 100 μg/mL streptomycin (GIBCO) in a humidified atmosphere of 5% CO_2_, respectively.

### 2.3 Peptide

Peptide GPC3_144‐152_ (Glu‐Tyr‐Ile‐Leu‐Ser‐Leu‐Glu‐Glu‐Leu, a purity of >98%) was synthetized by GL Biochem. We chose GPC3_144‐152_ as the antigen determinant because it has a higher immunogenicity than GPC3_298‐306_ by inducing stronger peptide‐specific CTL responses at a lower dose in human HLA‐A2 transgenic mice and having a higher affinity to HLA‐A2 molecules in humans.^20^, ^21^

### 2.4 Immunization with GPC3_144‐152_ peptide and isolation of splenocytes from HLA‐A2 transgenic mice

GPC3_144‐152_ in 7% NaHCO_3_ solution was mixed with 50% incomplete Freund's adjuvant (IFA, Sigma).^26^ Individual transgenic mice at 8‐week‐old were randomized and injected subcutaneously with multi‐points of the control IFA alone (blank control group, BC), 25, 50 or 100 μg GPC3_144‐152_/IFA as the low, medium or high dose group (*n* = 5 per group). Seven days later, the mice were boosted with the same dose of antigen. Ten days after boosting, three mice from each group were euthanized and their splenic lymphocytes were isolated.

### 2.5 Enzyme‐linked immunospot assay (ELISPOT)

The frequency of GPC3_144‐152_‐specific T cells was determined by ELISPOT assay using the ABCAM ELISPOT kit, according to the manufacturer's protocols. In brief, splenic lymphocytes (10^6^ cells/well) were isolated from immunized mice and treated in triplicate with 20 μg/mL GPC3_144‐152_ in 96‐well plates that had been coated with capture antibody against mouse IFN‐γ (Abcam) at 37°C for 12 h. After being washed, individual wells were added with horseradish peroxidase (HRP)‐conjugated IFN‐γ‐specific detection antibody and incubated at 4°C overnight. The bound antibodies were visualized with AEC. The spot form cells (SFC) in individual wells were analyzed using ImmunoSpot® S6 FluoroSpot Line (CTL). Similarly, the frequency of IFN‐γ‐secreting CTLs against TYST or control SW620 cells was determined after co‐cultured of CTLs with TYST or control SW620 cells for 72 h at the effector/target ratios (5:1, 10:1, and 20:1) in the presence or absence of a chosen dose of GPC3_144‐152_ peptide.

### 2.6 CD8^+^ T‐cell isolation and flow cytometric analysis

The remaining two mice in each group were euthanized and their splenic CD8^+^ T cells were isolated by magnetic separator using anti‐mouse CD8 microbeads isolation kit (Miltenyi Biotec). The purity of isolated CD8^+^ T cells were analyzed by flow cytometry using fluorescent anti‐CD8 (ThermoFisher) in a flow cytometer (ACCURI C6, BD).

### 2.7 Cell counting kit‐8 (CCK‐8) assay analysis of the cytotoxicity of primed CD8^+^ T cells against TYST cells in vitro

TYST cells (1 × 10^4^ cells/well) were cultured in 96‐well plates overnight as target cells (T) and co‐cultured with mouse splenic CD8^+^ T cells (E) from the mice that had been immunized with 50 μg GPC3_144‐152_ peptide at an E:T ratio of 10:1 in the presence of 2 × 10^−4^–2 × 10^2^ μg/mL GPC3_144‐152_ peptide for 72 h. During the last 1‐h culture, 20 μL CCK‐8 (LiankeBio) working solution was added into each well. The controls included TYST cells or CD8^+^ T cells alone. The absorbance of each well supernatant was measured at 450 nm in a microplate reader (SpectraMax Plus 384). The cytotoxicity of CD8^+^ T cells against TYST cells was calculated by the formula: Cytotoxicity (%) = [(Ac‐As)/(Ac‐Ab)] × 100% (As: Experimental OD; Ac: Control OD; Ab: Blank OD).

### 2.8 Establishment of cGAS stably silencing TYST‐sh‐cGAS cells

TYST cells were transfected with three cGAS‐specific siRNAs and a control scramble siRNA that were designed and generated by Ribo Biotech using ExFect Transfection Reagent (Vazyme). The efficacy of cGAS silencing was examined by RT‐qPCR and Western blot. Subsequently, the optimal siRNA sequence (GGCTATCCTTCTCTCACAT) was obtained for construction of a plasmid on pHBLV‐U6‐MCS‐PGK‐PURO vector, which was verified by PCR. After amplified, the plasmids of psPAX2 and pMD2G were transfected into 293 cells using Lipofiter™ (Hanbio Biotech) and the cellular supernatants were harvested for the purification of lentivirus virions. Finally, TYST cells were transduced with the control lentivirus or the lentivirus for cGAS shRNA expression at a MOI of 10 and cultured in 4 μg/mL puromycin for 7 days to generate cGAS stably silencing or control cells.

### 2.9 Co‐culture of CD8 ^+^ T cells with TYST or TYST‐sh‐cGAS cells

The purified splenic CD8^+^ T cells (4 × 10^5^ cells/well) from the mice that had been immunized with 50 μg GPC3_144‐152_ in IFA were co‐cultured with 2 × 10^4^ cells/well of TYST or TYST‐sh‐cGAS cells at an E:T ratio of 20 in the presence or absence of 0.0283 μg/mL of GPC3_144‐152_ for different time periods (4, 48 or 72 h). The peptide dose enhanced the cytotoxicity of CD8^+^ T cells by 50%, based on our preliminary studies. The suspended CD8^+^ T cells and adhered tumor cells were analyzed by flow cytometry assays, immunofluorescence or Western blot.

### 2.10 Flow cytometry assays

The different groups of cells were co‐cultured at 37°C for 4 h and after brief centrifugation, the CD8^+^ T cells were isolated. The isolated T cells were stained with APC‐anti‐CD8 (BD Biosciences) alone or APC‐anti‐CD8 and PE‐anti‐IFN‐γ (BD Biosciences) or BV421‐anti‐granzyme B (Abcam). The cells were analyzed by flow cytometry for the purity and their function on the CytoFlex analytic flow cytometer (Beckman‐Coulter), and the data were analyzed by Flowjo software (Tree Star). In addition, the adherent tumor cells were stained with antibodies, followed by immunofluorescence.

### 2.11 Western blot analysis

The different groups of cells were harvested and lyzed in RIPA buffer (Beyotime) containing protease and phosphatase inhibitors, followed by centrifugation. After quantitation of protein concentrations using the BCA Protein Assay Kit (Beyotime), the cell lysates (30 μg/lane) were separated by SDS‐PAGE and transferred onto PVDF membranes. The membranes were blocked in 5% skim milk in TBST and probed at 4°C overnight with primary antibodies against TBK1, p‐TBK1, IRF3, p‐IRF3 (1:1000, CST) and GAPDH (1:5000, LiankeBio). The bound antibodies were reacted with HRP‐conjugated secondary mouse or rabbit antibodies (LiankeBio) at room temperature for 1 h and visualized using enhanced chemiluminescence kit (ECL kit, Beyotime) in the gel imaging analyzer (ChemiDoc XRS + System, Bio‐RAD).

### 2.12 Immunofluorescence

The different groups of cells were cultured on glass coverslips in 24‐well plates and fixed with 4% paraformaldehyde for 20 min, permeabilized in 0.1% of Triton X‐100 for 20 min and blocked in 10% FBS for 1 h. The cells were probed at 4°C overnight with primary antibodies against p‐γH2AX (1:200, Abcam) or cGAS (1:200, Abcam). After being washed, the cells were incubated with fluorescent secondary antibodies, followed by nuclear‐staining with DAPI (Sigma). The cells were photoimaged under a confocal microscope at a 630× magnification (Model LSM880, Zeiss).

### 2.13 In vivo studies

Individual BALB/c (nu/nu) mice were inoculated subcutaneously with 5 × 10^6^ TYST‐control or TYST‐sh‐cGAS cells that had been mixed with 100 μL Matrigel into their left anterior axilla (*n* = 5 per group). When tumors reached an average volume of 200 mm^3^, the mice were randomized and treated with vehicle saline as the TYST group (A group), TYST sh‐cGAS group (B group), intravenously with 5 × 10^6^/0.1 mL NS‐CD8^+^ T as the TYST+NS‐CD8^+^ T group (C group), TYST sh‐cGAS+NS‐CD8^+^ T group (D group), or with the same dose of CD8^+^ T cells, together with 50 μg GPC3_144‐152_ as the TYST+GPC3‐CD8^+^ T group (E group) and TYST‐sh‐cGAS+GPC3‐CD8^+^ T group (F group), respectively. Five days later, the mice were treated with the same reagents again. The dynamic growth and body weights of individual mice were measured daily up to 10 days post the first treatment. The mice were euthanized and their tumor tissues were dissected and photoimaged. Tumor volumes were calculated by the equation $V={\mathrm{ab}}^{2}/2$ (a and b for the maximal and minimal diameter in millimeters, respectively).

### 2.14 Immunohistochemistry (IHC) and apoptosis detection (TUNEL)

Individual tumor specimens were fixed in 10% of formalin and paraffin‐embedded. The tumor tissue sections (4 μm) were dewaxed, rehydrated, and subjected to antigen retrieval. The sections were incubated with specific primary antibodies against CD8 (Protintech, 1:800), granzyme B (Bioss, 1:100), IFN‐β (Bioss, 1:100) and glypican‐3 (Abcam, 1:200) at 4°C overnight. After being washed, the sections were reacted with HRP‐conjugated second antibodies and visualized with DAB. IHC signals were photoimaged (200 × magnification) under a light microscope (BX43 type, OLYMPUS). In addition, the frequency of apoptotic cells in tumor sections was measured by TUNEL assay using the TUNEL Detection Kit (ROCHE), according to the manufacture instructions.

### 2.15 Statistical analysis

Data are presented as means ± standard deviation (SD) of each group from three or five independent experiments and were analyzed using GraphPad Prism version 9.4.1. The comparison of three or more groups of unpaired variables was performed by one‐way ANOVA and post hoc Tukey test or the Games‐Howell test. The difference between two variables was analyzed by two‐way ANOVA and post hoc Tukey test. The data were considered statistically significant when a *p*‐value of <0.05.

## 3 RESULTS

### 3.1 Immunization with GPC3_144_ _‐152_ peptide induces potent antigen‐specific CD8 ^+^ T‐cell responses in vivo

**FIGURE 1:**
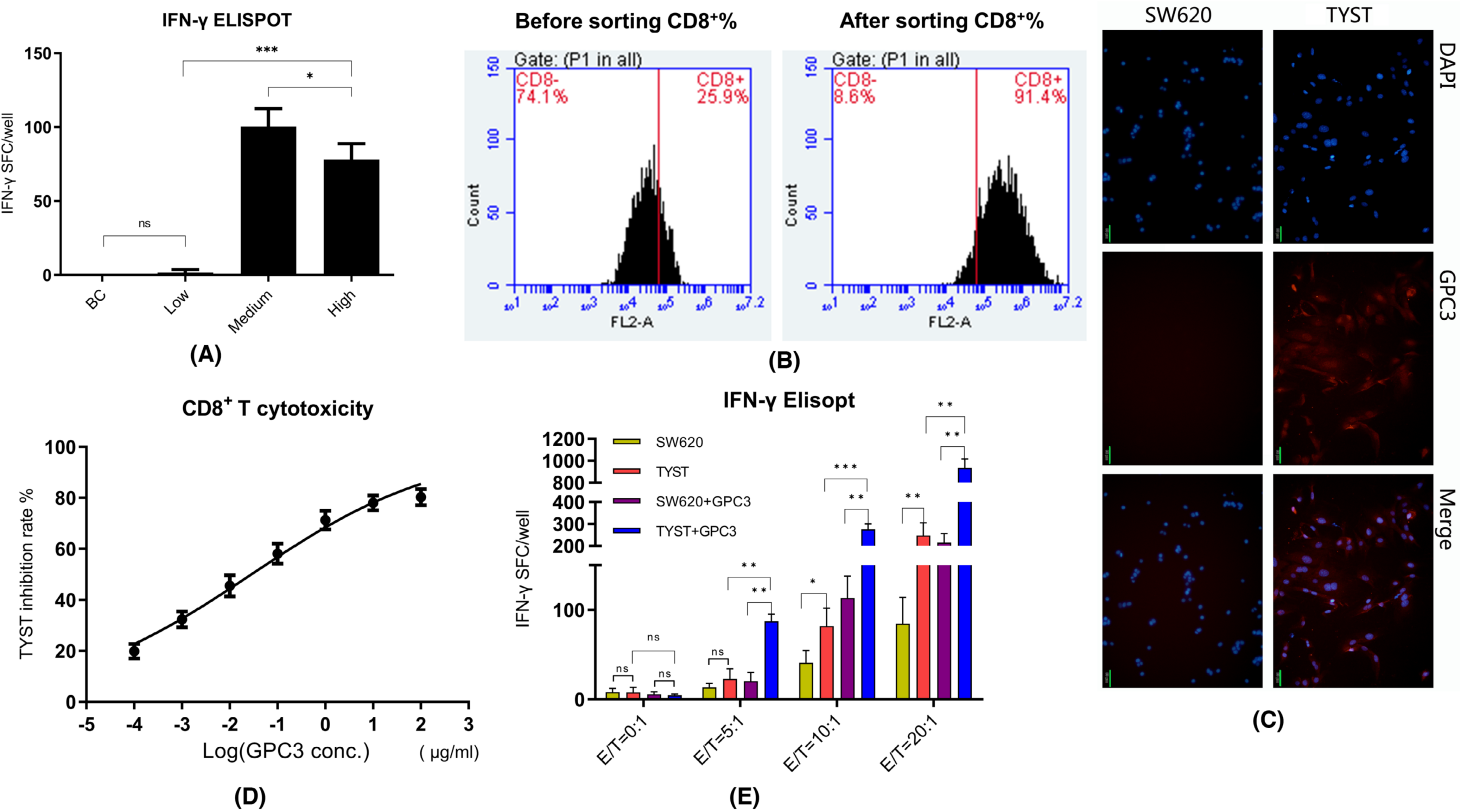
Induction and acquisition of GPC_144‐152_‐specific CD8^+^ T cells in vivo and detection of antigen‐specific CD8^+^ T cell cytotoxicity against TYST cells in vitro. (A) Following immunization with the indicated doses of GPC_144‐152_ peptide twice, splenic lymphocytes were isolated from the different groups of mice (*n* = 3 per group) and their responses to the peptide were tested by ELISPOT assays. Data are from three separate experiment. BC, Blank control group in the absence of antigen peptide. (B) CD8^+^ T cells from the indicated group of mice (immunized with 50 μg GPC_144‐152_ peptide) were purified by positive selection using microbeads, and analyzed by flow cytometry. Data are representative flow cytometry histograms. (C) The expression of GPC3 in TYST cells or SW620 cells (negative control) was detected by immunofluorescent assay. Data are representative images from each type of cells. Scale bars, 50 μm. (D) Antigen‐specific CD8^+^ T cells were co‐cultured with TYST cells at a ratio of 10:1 in the presence of the indicated concentrations of antigen peptide for 72 h. Proliferative responses were determined by CCK‐8 assays. (E) The purified CD8^+^ T cells were mixed with TYST or SW620 cells at the indicated ratios in the presence or absence of GPC3_144‐152_ for 72 h to test their IFN‐γ responses by ELISPOT assays. Data are represented as the mean ± SD. SFC, spot forming cells; *p < 0.05; **p < 0.01; ***p < 0.001; ns, none significance.

To determine the immunogenicity of GPC3_144‐152_ peptide, male B‐HLA‐A2.1 transgenic mice were immunized with different doses of GPC3_144‐152_ peptide in IFA twice and 10 days later, their splenic lymphocytes were isolated for testing CD8^+^ T‐cell responses to GPC3_144‐152_ peptide by IFN‐γ‐based ELISPOT assays ex vivo. There was no detectable T‐cell response to the bank control (BC) mice and barely few spot forming cells in the mice vaccinated with a low dose (25 μg) of GPC3_144‐152_ peptide in IFA (Figure 1A). However, strong T‐cell responses to the peptide were detected in the mice vaccinated with a medium dose (50 μg) of GPC3_144‐152_ peptide in IFA and the number of IFN‐γ‐secreting T cells in the medium group was significantly greater than those in the high group of mice. Hence, vaccination with a medium dose of the peptide in IFA induced stronger T‐cell responses in B‐HLA‐A2.1 transgenic mice.

### 3.2 GPC3_144_ _‐152_ peptide induces CTLs selectively against tumor cells

Next, we isolated CD8^+^ T cells from the mice that had been vaccinated with a medium dose of GPC3_144‐152_ peptide using microbeads and following APC‐anti‐CD8 staining, we analyzed the purity of CD8^+^ T cells by flow cytometry. The isolated cells had a purity of >90% (Figure 1B). Simultaneously, we analyzed the expression of GPC3 in TYST and control SW620 cells by immunofluorescence. The results displayed that GPC3 was expressed by TYST cells, consistent with a previous observation,^16^ but not in the control SW620 cells (Figure 1C).

Furthermore, we evaluated the efficacy of CTLs induced by peptide vaccination against TYST cells. We co‐cultured CTLs with TYST cells at an E:T ratio of 10 in the presence of different concentrations of GPC3_144‐152_ peptide for 72 h in vitro. We determined the cytotoxicity of CTLs against TYST cells by CCK‐8 assays. As shown in Figure 1D, the cytotoxicity of CTLs against TYST cells appeared to depend on the concentrations of GPC3_144‐152_ peptide. Similarly, we employed ELISPOT assays to determine the frequency of IFN‐γ‐secreting CTLs against TYST or control SW620 cells in the presence or absence of a chosen dose of GPC3_144‐152_ peptide. We found that compared with SW620 or TYST cells without GPC3_144‐152_ peptide, addition of GPC3_144‐152_ peptide into the co‐cultures with different E/T ratios increased the frequency of IFN‐γ‐secreting CTLs against SW620 and TYST cells in an effector‐dependent manner (Figure 1E). As expected, the frequency of TYST‐reactive IFN‐γ‐secreting CD8^+^ T cells in the co‐culture system was significantly higher than that of SW620‐reactive CD8^+^ T cells. Moreover, the addition of GPC3_144‐152_ peptide into the SW620 co‐culture system further activates CTLs, suggesting that HLA‐A2.1 molecules on either SW620 or TYST cells may effectively present GPC3_144‐152_ peptide to activate its specific CTLs in vitro.

### 3.3 Adoptively transferred CD8 ^+^ T cells inhibit tumor growth in mice

**FIGURE 2:**
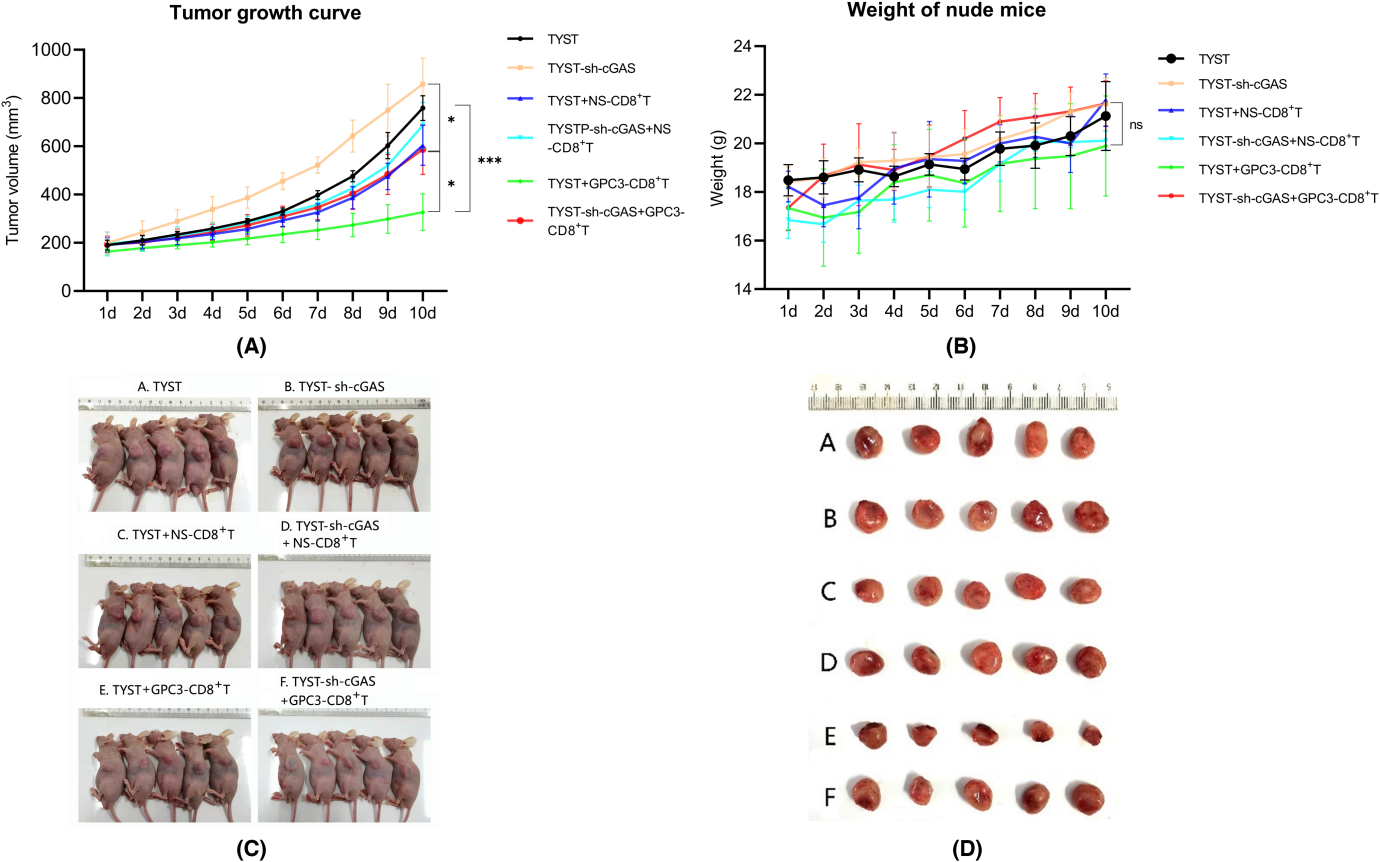
Adoptive transfer of GPC3 peptide‐specific CD8^+^ T cells and treatment with GPC3_144‐152_ significantly inhibited the growth of TYST tumors, but less effective for the cGAS‐silenced TYST tumors in mice. BALB/C nude mice were randomized and inoculated subcutaneously with TYST or cGAS‐silenced TYST cells. When the tumor reached at 200 mm^3^, these mice were treated intravenously with vehicle, CD8^+^ T cells alone (NS‐CD8^+^ T cells), or CD8 ^+^ T cells plus peptide. The mice were monitored for tumor growth and body weights longitudinally. (A) The dynamic growth of the different groups of tumors (*n* = 5 per group). (B) The body weights of the different groups of mice. (C) The presence of subcutaneous tumors in tumor‐bearing mice after 10 days of treatment in different groups. (D) The tumor tissues were harvested on day 10. A, TYST; B, TYST‐sh‐cGAS; C, TYST+NS‐CD8^+^ T; D, TYST‐sh‐cGAS+NS‐CD8^+^ T; E, TYST+GPC3‐CD8^+^ T; F, TYST‐sh‐cGAS+GPC3‐CD8^+^ T. *p < 0.05; ***p < 0.001; ns, none significance.

It has been reported that activation of the cGAS/STING signaling in the tumor cells promotes the recruitment of CTLs into the tumor microenvironment and can enhance the antitumor responses of CTLs.^27^ To explore the potential antitumor effect of CTLs from GPC3_144‐152_ peptide‐vaccinated mice and the impact of the cGAS/STING signaling on the inhibition of CTLs on the growth of TYST tumors in vivo, BALB/c nude mice were inoculated subcutaneously with TYST‐control or TYST‐sh‐cGAS cells into their left anterior axilla to establish a xenograft TYST. When tumors reached an average volume of 200 mm^3^, the mice were randomized and treated vehicle saline as the TYST group (A group), TYST sh‐cGAS group (B group), intravenously with 5 × 10^6^ CD8^+^ T cells from GPC3_144‐152_‐vaccinated mice as the TYST‐CD8^+^ T group (C group), TYST‐sh‐cGAS‐CD8^+^ T group (D group), or the same dose of CD8^+^ T cells, together with GPC3_144‐152,_ as the TYST‐GPC3‐CD8^+^ T group (E group) and TYST‐sh‐cGAS‐GPC3‐CD8^+^ T group (F group). Their tumor growth and body weights were monitored longitudinally up to 10 days post the first treatment (Figure 2A,B). First, there was no significant difference in body weights among the different groups of mice. Second, the tumor volumes in the TYST‐sh‐cGAS group were obviously larger than that of other groups. Furthermore, while adoptive transfer of CD8^+^ T cells alone did not significantly alter the growth of implanted tumors adoptive transfer of CD8^+^ T cells, together with GPC3_144‐152_ peptide, significantly decreased the tumor volumes in the TYST‐sh‐cGAS and TYST groups of mice. Moreover, adoptive transfer of CD8^+^ T cells together with GPC3_144‐152_ peptide also significantly reduced the tumor volumes of the TYST group, compared with those in the TYST‐sh‐cGAS group (*p* = 0.0189). Similar patterns of tumor sizes were observed in the different groups of mice (Figure 2C,D). These support the notion that activation of the cGAS/STING signaling in the tumors is crucial for recruiting antitumor CTLs into the tumor environment to inhibit the growth of tumors.^28^

### 3.4 Adoptively transferred CTLs infiltrate into the TYST microenvironment and induce tumor cell apoptosis in vivo

**FIGURE 3:**
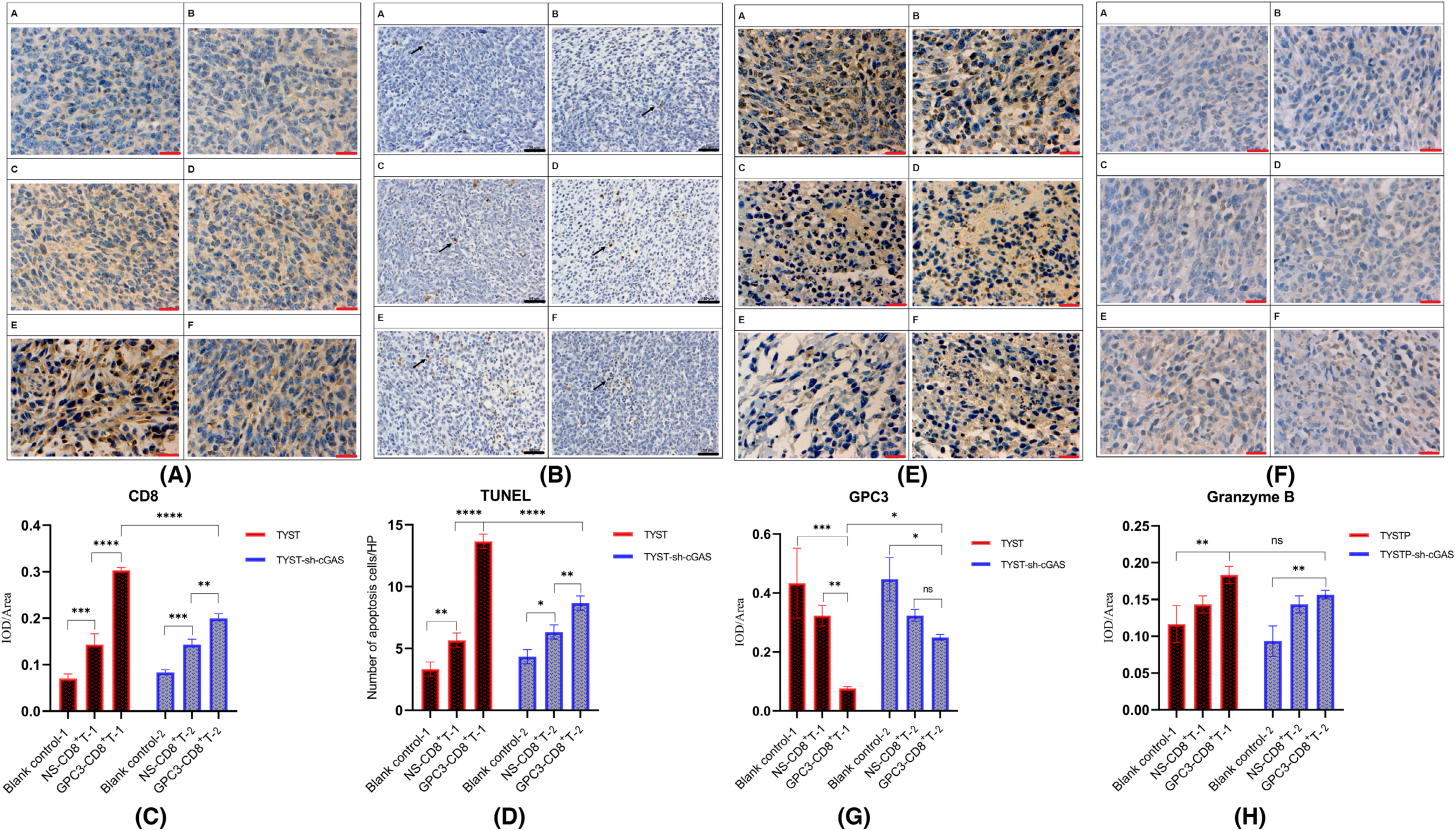
Immunohistochemical and TUNEL analysis of CD8, GPC3, granzyme B expression and apoptotic cells in the tumors from different groups of mice. Ten days after treatment with CD8^+^ T cells and GPC3_144‐152_ peptide, the mice were euthanized and their tumors were dissected for IHC and TUNEL assays. Data are representative images or presented as the mean ± SD of each group (*n* = 3) from three separate experiments. (A, C) IHC and quantitative analysis of CD8 expression; (B, D) TUNEL images and quantitative analysis of apoptotic cells. (E, G) IHC and quantitative analysis of GPC3 expression; (F,H) IHC and quantitative analysis of granzyme B expression. A: TYST (Blank control‐1); B: TYST‐sh‐cGAS (Blank control‐2); C: TYST+NS‐CD8^+^ T cells (NS‐CD8 ^+^ T cells‐1); D: TYST‐sh‐cGAS+NS‐CD8 ^+^ T cells (NS‐CD8^+^ T cells‐2); E: TYST+GPC3‐CD8 ^+^ T cells (GPC3‐CD8 ^+^ T cells‐1); F: TYST‐sh‐cGAS+GPC3‐CD8 ^+^ T cells (GPC3‐CD8 ^+^ T cells‐2). Scale bars, 25 (red)–50 (black) μm. Magnification× 200. *p < 0.05, **p < 0.01, ***p < 0.001, ****p < 0.0001; ns, none significance.

To understand the mechanisms underlying the effect of antitumor CTLs, we analyzed T lymphocyte infiltration (TIL) and tumor cell apoptosis in the tumor microenvironment by IHC and TUNEL assays. The results displayed that there were many CD8^+^ T‐cell infiltrates in the tumor microenvironment of the TYST‐GPC3‐CD8^+^ T group, but the numbers of CD8^+^ T‐cell infiltrates in the TYST‐sh‐cGAS+GPC3‐CD8^+^ T group were significantly higher than those in any of the other groups, except for the E group (*p* < 0.05, Figure 3A,C). On the other hand, there were many more apoptotic tumor cells in tumor tissue of mice that had been adoptively transferred with CD8^+^ T cells, together with GPC3_144‐152_ peptide than those from the mice that had been adoptively transferred with CD8^+^ T cells alone (Figure 3B,D). Similarly, the number of apoptotic tumor cells in the TYST‐sh‐cGAS+GPC3‐CD8^+^ T group was significantly less than those in the TYST‐GPC3‐CD8^+^ T group (*p* < 0.0001). Together, these data suggest that cGAS silencing may inhibit the infiltration of adoptive transferred CTLs into the tumor environment to trigger tumor cell apoptosis.

Moreover, to evaluate the killing effect of CTLs on TYST, the levels of GPC3 and granzyme B expression were also determined by IHC in tumor tissues. There were similar levels of GPC3 expression between the TYST and TYST‐sh‐cGAS tumors in the absence of any treatment, indicating that cGAS silencing did not affect the expression of GPC3 in TYST (Figure 3E,G). Similar levels of GPC3 expression were observed in the TYST and TYST‐sh‐cGAS tumors from the mice that had been adoptively transferred with CD8^+^ T cells alone. Apparently, the adoptively transferred CD8^+^ T cells in the absence of peptide treatment may not allow sufficient CTLs to enter the tumor microenvironment for killing TYST cells. It was notable that while adoptive transfer of CD8^+^ T cells and treatment with GPC3 peptide significantly decreased the levels of GPC3 expression in TYST tumors the same treatments only slightly, but insignificantly, decreased GPC3 expression in the TYST‐sh‐cGAS tumors. Thus, treatment with GPC3_144‐152_ peptide may effectively promote the infiltration of adoptively transferred CD8^+^ T cells into the TYST tumors, but less efficiently in the TYST‐sh‐cGAS tumors. The levels of granzyme B expression usually reflect the potency of CTL cytotoxicity against tumor cells. In contrast, the levels of granzyme B expression were significantly higher in the tumors from the GPC3‐CD8^+^ T‐cell group of mice than that in the blank control group (Figure 3F,H, *p* < 0.01), but there was no statistical difference in the levels of granzyme B expression between the tumors with CD8^+^ T‐cell treatment alone and blank control groups in either TYST or TYST‐sh‐cGAS tumor models, also between the tumors with CD8^+^ T‐cell treatment alone and GPC3‐CD8^+^ T‐cell groups (*p* > 0.05). These results suggested that the cytotoxic effect of CTLs against tumor might not be mainly mediated by the granzyme B, but might act through other pro‐apoptotic pathways to induce TYST cell apoptosis in the tumor environment.

### 3.5 GPC3_144_ _‐152_ peptide enhances the antitumor effect of CTLs in vitro

**FIGURE 4:**
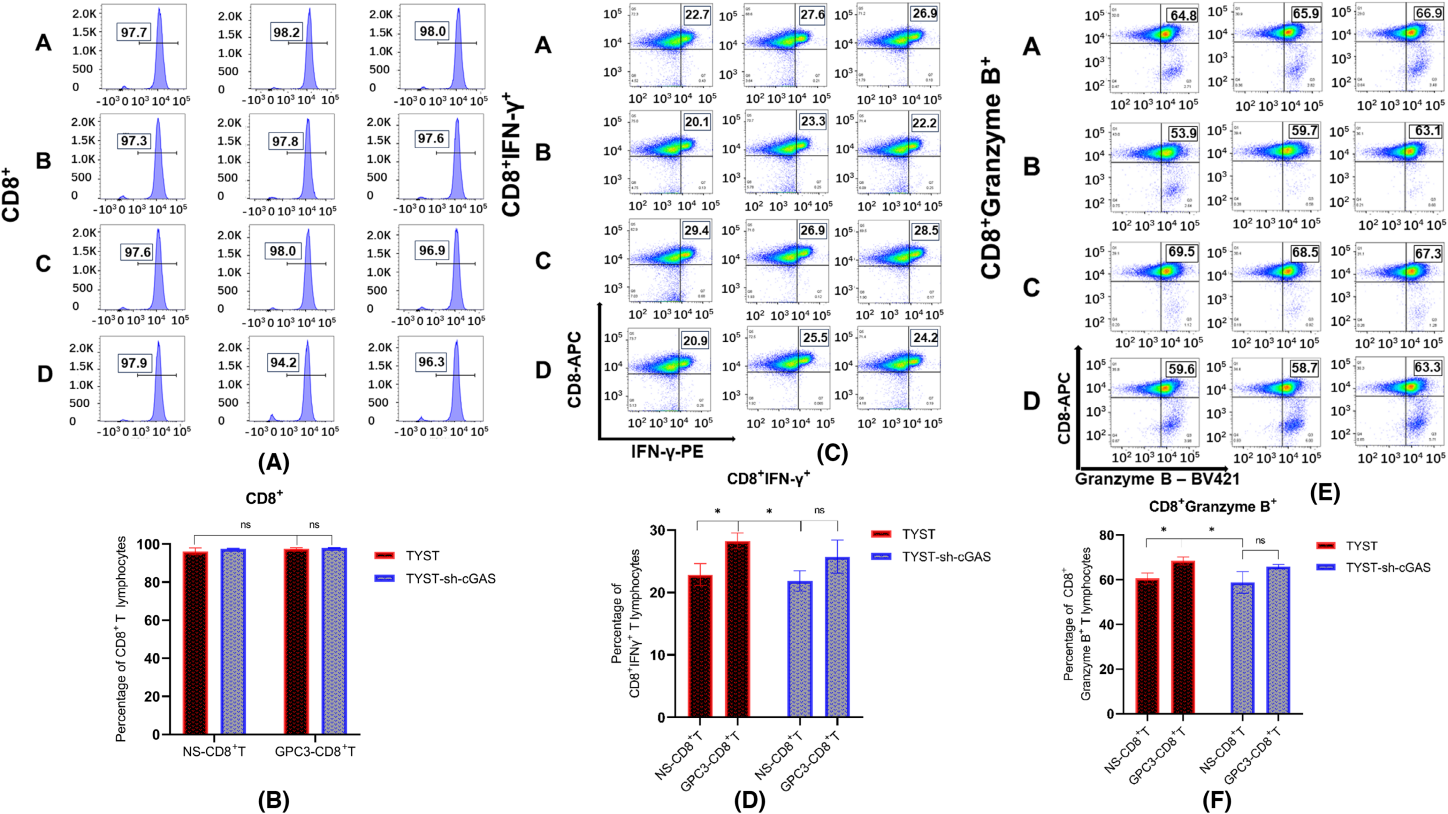
GPC3_144‐152_ peptide enhances the cytotoxicity of CTLs against TYST cells in vitro. The purified CD8 ^+^ T cell were co‐cultured with TYST or TYST‐sh‐cGAS cells at a ratio of 20:1 in the presence or absence of 0.0283 μg/mL GPC3_144‐152_ peptide for 4 h. The percentages of CD8^+^ T cells, IFN‐γ^+^ or granzyme B^+^ CD8^+^ T cells were analyzed by flow cytometry. Data are representative flow cytometry charts and histograms and quantitative mean ± SD of each group (*n* = 3) from three separate experiments. (A, B) The flow cytometry charts and quantitative analysis of CD8 expression. (C, D) The flow cytometry charts and quantitative analysis of CD8^+^IFN‐γ^+^ expression. (E, F) The flow cytometry charts and quantitative analysis of CD8^+^Granzyme B^+^. *p < 0.05; ns, none significance. A, TYST‐sh‐cGAS+GPC3‐CD8^+^ T; B, TYST‐sh‐cGAS+NS‐CD8^+^ T; C, TYST+GPC3‐CD8^+^ T; D, TYST+NS‐CD8^+^ T.

To further evaluate whether GPC3_144‐152_ peptide could enhance the cytotoxicity of CTLs against TYST cells in vitro, we co‐cultured CD8^+^ T cells with TYST or TYST‐sh‐cGAS cells at an E:T ratio of 20:1 in the presence or absence of a chosen dose of GPC3_144‐152_ peptide for 4 h. Flow cytometry analyses revealed that there was no significant difference in the percentages of CD8^+^ T cells regardless of cultured with TYST and TYST‐sh‐cGAS cells in the presence or absence of GPC3_144‐152_ peptide in the co‐culture system (Figure 4A,B). This indicated that co‐culture of CD8^+^ T cells with peptide‐loaded tumor cells for a short period failed to stimulate T‐cell proliferation or apoptosis in our experimental system. However, compared with those with CD8^+^ T‐cell treatment alone, addition of GPC3_144‐152_ peptide significantly increased the frequency of IFN‐γ‐secreting and granzyme B‐secreting CD8^+^ T cells that had been co‐cultured with TYST, but not with TYST‐sh‐cGAS cells (Figure 4C–F). This indicated that the peptide loaded on the wild‐type, but not the cGAS silencing, tumor cell surface enhanced the activity of effector/memory CD8^+^ T cells in vitro, suggesting that cGAS silencing in the target tumor cells might hinder the further activation of CD8^+^ T cells.

### 3.6 Addition of GPC3_144_ _‐152_ peptide enhances the CTL‐activated cGAS signaling in the TYST cells

**FIGURE 5:**
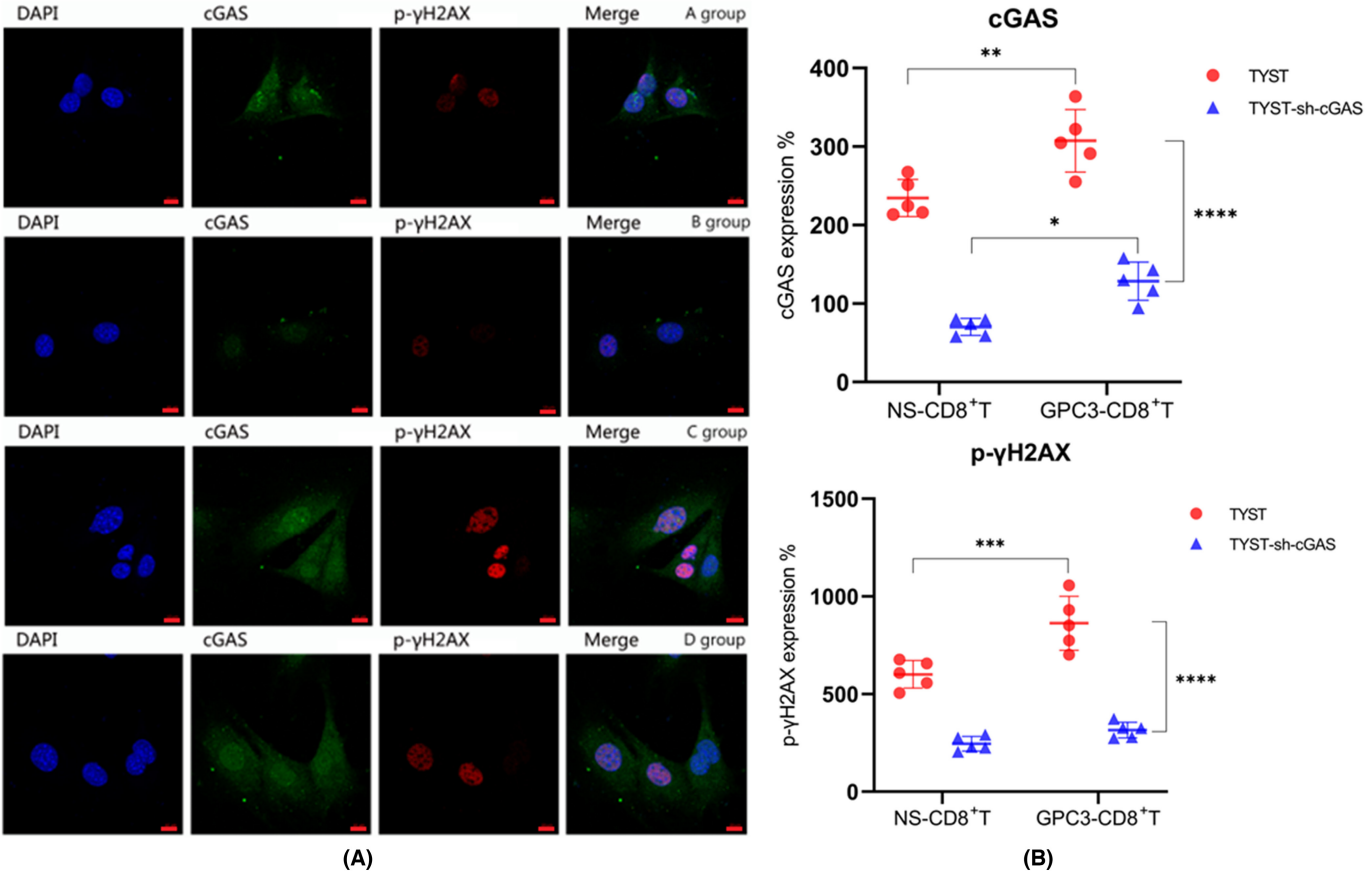
Addition of GPC3_144‐152_ peptide into the co‐culture of CD8^+^ T cells with TYST or TYST‐sh‐CGAS cells enhances cGAS expression and γH2AX phosphorylation in tumor cells. Following co‐culture of CD8^+^ T cells with TYST or TYST‐sh‐cGAS cells at a ratio of 20:1 for 72 h in the presence or absence of 0.0283 μg/mL GPC3_144‐152_ peptide. The average fluorescence intensity expression of cGAS and p‐γH2AX in tumor cells were quantified by immunofluorescence. Data are representative images (magnification x 630) or quantitative mean ± SD of each group (*n* = 5). (A) Immunofluorescent images of cGAS^+^ or p‐γH2AX^+^ tumor cells. DAPI (blue)‐, cGAS (green)‐ and p‐γH2AX (red)‐stained cells. Scale bar, 10 μm. (B) Quantitative analysis of the expressions of p‐γH2AX and cGAS in tumor cells. *p < 0.05, **p < 0.01, ***p < 0.001; ****p < 0.0001. A, TYST sh‐cGAS+GPC3‐CD8^+^ T; B, TYST sh‐cGAS+NS‐CD8^+^ T; C, TYST+GPC3‐CD8^+^ T; D, TYST+NS‐CD8^+^ T.

**FIGURE 6:**
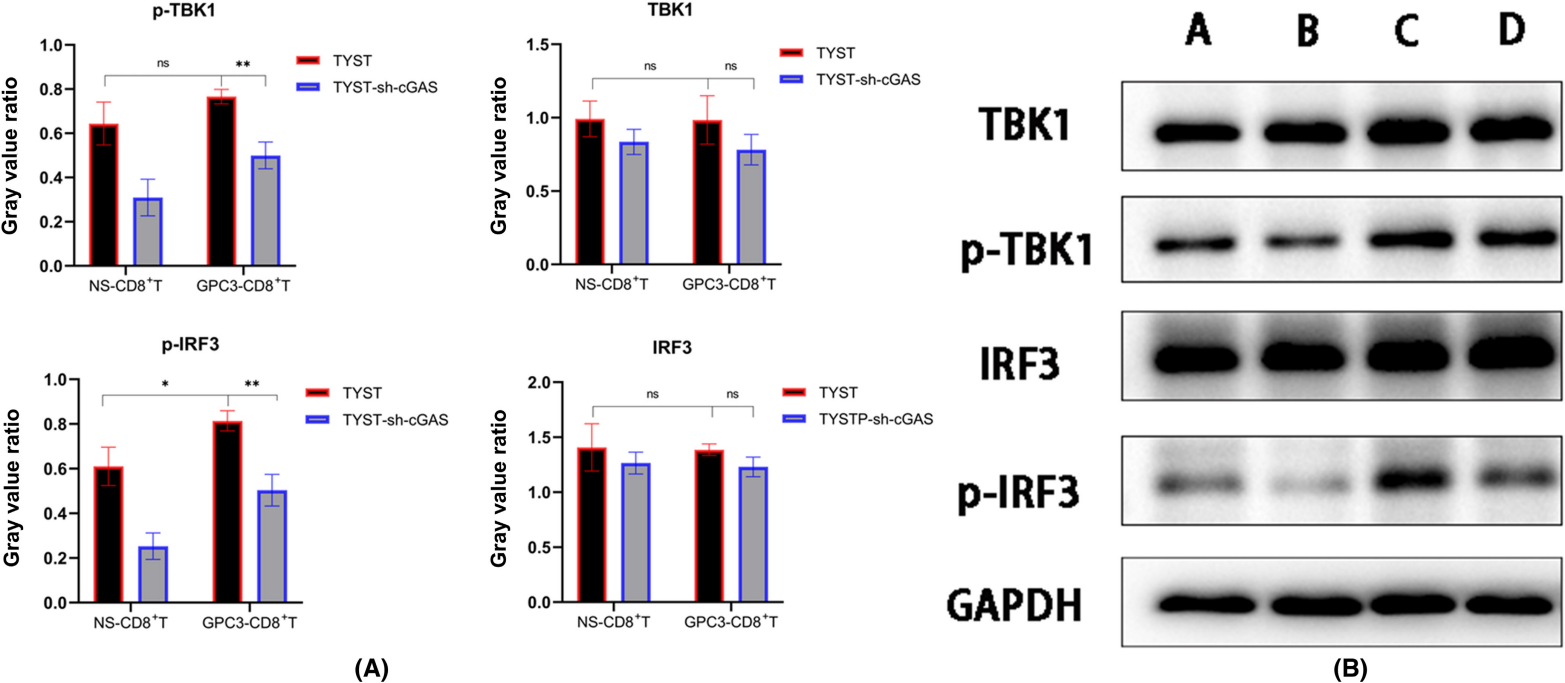
Addition of GPC3_144‐152_ peptide into the co‐culture of CD8^+^ T cells with TYST or TYST‐sh‐cGAS cells enhances TBK1 and IRF3 phosphorylation in tumor cells. CD8^+^ T cells were co‐cultured with TYST or TYST‐sh‐cGAS cells at a ratio of 20:1 in the presence or absence of 0.0283 μg/mL GPC3_144‐152_ peptide for 48 h. The adherent cells were isolated and tested for the relative levels of TBK1 and IRF3 expression and phosphorylation in the different groups of tumor cells by Western blot. Data are representative images or expressed as the mean ± SD of each group from three independent experiments. (A) The ratio of gray value of each protein to the gray value of the internal control. (B) Representative electophoretic images of each protein molecule. *p < 0.05, **p < 0.01; ns, none significance. A, TYST‐sh‐cGAS+GPC3‐CD8^+^ T; B, TYST‐sh‐cGAS+NS‐CD8^+^ T; C, TYST+GPC3‐CD8^+^ T; D, TYST+NS‐CD8^+^ T.

A growing evidence has shown that there is a spontaneous inflammtory response against the tumor and there are many inflammatory infiltrates in human tumor microenvironment during the process of tumor progression.^29^, ^30^ The infiltrated CD11c^+^ dendritic cells (DCs) can secrete Type I IFNs to induce adaptive immune responses, which are positively regulated by the cGAS/STING signaling in the tumors. Next, we tested whether the enhanced CTL responses to GPC3_144‐152_ peptide could modulate the cGAS/STING signaling in TYST cells. Following co‐culture of CD8^+^ T cells with TYST or TYST‐sh‐cGAS cells in the presence or absence of GPC3_144‐152_ peptide, we analyzed the levels of cGAS and γH2AX phosphorylation by immunofluorescence, and the relative levels of TANK‐binding kinase 1 (TBK1) and interferon regulatory factor 3 (IFR3) expression and phosphorylation in tumor cells by Western blot. Immunofluorescent confocal analyses revealed that compared with those co‐cultured with TYST cells, the levels of cGAS and γH2AX phosphorylation decreased in TYST‐sh‐cGAS cells that had been co‐cultured with CD8^+^ T cells. Addition of GPC3_144‐152_ peptide significantly elevated the levels of cGAS expression in TYST or TYST‐sh‐cGAS cells that had been co‐cultured with CD8^+^ T cells (Figure 5A,B). Meanwhile, there was no significant difference in the relative levels of TBK1 and IFR3 expression between TYST and TYST‐sh‐cGAS cells regardless of the presence or absence of GPC3_144‐152_ peptide (Figure 6A,B). However, the relative levels of TBK1 phosphorylation in TYST cells were significantly higher than that in the TYST‐sh‐cGAS cells regardless of the presence of GPC3_144‐152_ peptide. Similarly, the relative levels of IFR3 phosphorylation in the TYST cells were also significantly higher than that in the TYST‐sh‐cGAS cells and addition of GPC3_144‐152_ peptide significantly increased the relative levels of IFR3 phosphorylation in both TYST and TYST‐sh‐cGAS cells.

**FIGURE 7:**
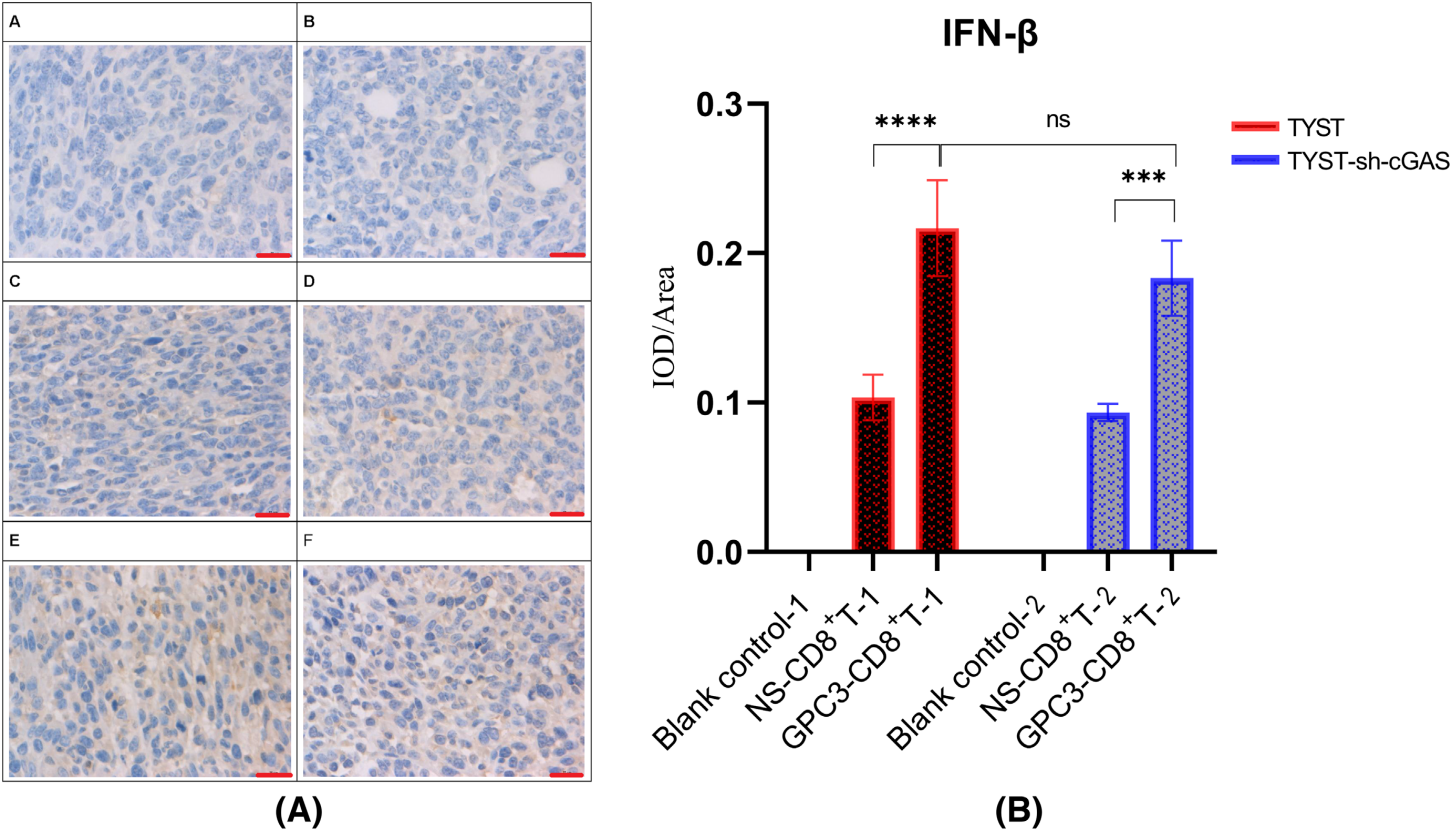
Treatment with GPC3_144‐152_ peptide increases IFN‐β expression in the tumors. BALB/c nude mice were inoculated subcutaneously with TYST or TYST‐sh‐cGAS cells. When the tumors reached at 200 mm^3^, the mice were randomized and treated with vehicle (blank control) or CD8^+^ T cells with or without GPC3_144‐152_ peptide for twice. At 10 days post treatment, the mice euthanized and their tumor tissues were dissected and subjected to IHC analysis of IFN‐β expression. Data are representative images or expressed as the mean ± SD of each group (*n* = 3) from three separate experiments. (A) Immunohistochemical expression of IFN‐β in each treatment group. (B) Quantitative analysis of IFN‐β expression in different treatment groups. A: TYST (Blank control‐1); B: TYST‐sh‐cGAS (Blank control‐2); C: TYST+NS‐CD8^+^ T cells (NS‐CD8^+^ T cells‐1); D: TYST‐sh‐cGAS+NS‐CD8^+^ T cells (NS‐CD8^+^ T cells‐2); E: TYST+GPC3‐CD8^+^ T cells (GPC3‐CD8^+^ T cells‐1); F: TYST‐sh‐cGAS+GPC3‐CD8^+^ T cells (GPC3‐CD8^+^ T cells‐2). Scale bars, 25 μm. ***p < 0.001; ****p < 0.0001; ns, none significance.

Finally, we characterized the expression of IFN‐β in the tumor environment of different groups of tumors by IHC. First, there was little IFN‐β in both the TYST and TYST‐sh‐cGAS tumors from the blank control mice without receiving adoptively transferred CTLs (Figure 7A,B). The levels of IFN‐β expression in the tumors from the mice that had been adoptively transferred CD8^+^ T cells clearly higher than those in the tumors from the blank control mice without CD8^+^ T‐cell treatment. Moreover, the levels of IFN‐β expression in the tumors from the mice that had been adoptively transferred CD8^+^ T cells and treated with GPC3_144‐152_ peptide were dramatically higher than those without peptide treatment, particularly in the TYST tumors. Together, the results indicate that tumor cell cytosolic detection of DNA by cGAS catalyzes the generation of 2′–5′ cyclic GMP‐AMP (cGAMP), which binds to STING, allowing it to recruit, phosphorylate, activate TBK1 and IRF3, and produce Type I IFNs.

## 4 DISCUSSION

Cancer vaccines are highly unique and attractive approaches to effective therapies for cancers by inducing antitumor immunity, which relies on identification of tumor‐specific antigens and innate signals provided by antigen presenting cells (APCs) to stimulate tumor‐specific T‐cell responses to tumor cells.^31^ In this study, we provided evidence that treatment with GPC3_144‐152_ peptide induced and enhanced effector/memory CD8^+^ T‐cell responses to TYST by activating the cGAS/STING signaling in tumor cells.

Tumor antigens that can be recognized and attacked by host's adaptive immune responses are essential to effective antitumor immunotherapy. GPC3, as a carcinoembryonic antigen, is an ideal target for antitumor immunotherapy, because its expression is up‐regulated in many types of pediatric solid tumors, especially in YSTs.^6^, ^18^ Previous studies have shown that vaccination with GPC3_144‐152_ peptide induces potent CD8^+^ T‐cell immunity against various types of cancers in rodents and in clinical trials.^8^, ^32^ Similarly, we observed that vaccination with a medium dose of GPC3_144‐152_ peptide in 50% IFA induced potent CD8^+^ T‐cell responses in human HLA‐A2.1 transgenic mice, consistent with a previous study.^33^ However, we observed that vaccination with a higher dose of the peptide only stimulated a moderate CD8^+^ T‐cell response, similar to a previous report.^34^ Apparently, it is not the case that the higher the dose of the antigenic vaccine, the more tumor‐specific T cells can be acquired and/or activated, also consistent with a previous viewpoint.^35^

Adoptive transfer of effector cells is an effective strategy for antitumor immunotherapy.^36^ In this study, we found that adoptive transfer of the purified CD8^+^ T cells from the mice that had been vaccinated with GPC3_144‐152_ peptide and treatment with the peptide significantly inhibited the growth of TYST tumors by increasing granzyme B‐secreting CD8^+^ T cells and the frequency of TUNEL^+^ apoptotic tumor cells in vivo. Similarly, treatment with GPC3_144‐152_ peptide also enhanced the cytotoxicity of CD8^+^ T cells against TYST cells in vitro. Hence, treatment with both antigenic peptide and CD8^+^ T cells was more effective than adoptive transfer of CD8^+^ T cells alone. The increased immunotherapeutic efficacy by peptide treatment may stem from that the treated peptide presented by APCs or tumor cells stimulated antigen‐specific T‐cell expansion or up‐regulated target antigen density on tumor cell surface to enhance their sensitivity to antitumor responses. Actually, treatment with the peptide enhanced CD8^+^ T‐cell immunity against TYST cells in a dose‐dependent manner in a given condition and more importantly, this therapeutic strategy was relatively safe as we did not observe that treatment with both CD8^+^ T cells and antigenic GPC3_144‐152_ peptide significantly altered the acting behaviors and body weights in mice, similar to a previous report.^26^ Of course, antitumor immunotherapy is usually needed to combine with other therapies, such as surgical resection of tumors, neoadjuvant or post‐operative chemotherapy or radiotherapy.

Antigen‐specific CD8^+^ T‐cell migration into the tumor is key for effectively killing tumor cells, although the number and activity of antigen‐specific CD8^+^ T cells can be significantly enhanced by peptide vaccines and adoptive transfer.^8^, ^37^ In the present study, we found that treatment with GPC3_144‐152_ peptide, together with adoptive transfer of the primed CD8^+^ T cells significantly increased the levels of CD8 and granzyme B expression in the TYST tumors. These indicated that treatment with the peptide increased the number of effector/memory CD8^+^ T cells in the tumor environment (TME) although we did not test other activation/effector markers in the TME. Furthermore, co‐culture of CD8^+^ T cells with TYST cells in the presence of GPC3_144‐152_ peptide did not change the percentages of CD8^+^ T cells among those groups, but significantly enhanced the frequency of IFN‐γ^+^CD8^+^ and granzyme‐B^+^CD8^+^ T cells following co‐cultured with TYST, but not with cGAS‐silenced TYST. These further support the notion that addition of antigen determinant enhances antigen‐specific CD8^+^ T‐cell activity. Therefore, the antitumor effects of CTLs in vivo seemed to be inconsistent with that in vitro. The difference may stem from a short period of experiments in vitro. Previous studies have shown that the degrees of antigen‐stimulated CD8^+^ T‐cell activation and proliferation are not only regulated by the antigen concentrations, but also by the stimulation time.^35^, ^38^ Unfortunately, we did not extend the experimental time periods to analyze the effects of GPC3_144‐152_ peptide on CD8^+^ T cells in the co‐culture system. Interestingly, GPC3, either a tumor suppressor or an oncofetal protein, can stimulate or inhibit cell proliferation, depending on its relevant signaling pathways,^10^, ^39^ and the decreased GPC3 expression may reflect tumor cell death or activating the cGAS/STING signal‐dependent TNF‐mediated necroptosis,^40^ or reprogramming of TYST cells caused by changes in the TME.^41^, ^42^, ^43^ We are interested in further investigating the molecular mechanisms underlying the action of antigen peptide treatment in enhancing CD8^+^ T‐cell‐mediated antitumor immunotherapy.

Type I IFNs (main subtypes, IFN‐α and β) are essential for priming tumor‐specific T‐cell responses and recruiting them into the TME,^30^, ^44^ and are primarily produced by DCs in the TME^45^ although they can also be secreted by tumor cells and other types of cells.^46^, ^47^ We found that treatment with GPC3_144‐152_ peptide, together with adoptive transfer of CD8^+^ T cells, significantly up‐regulated intratumoral IFN‐β expression, which should recruit and expand cytotoxic T cells.^30^ The up‐regulated IFN‐β expression may be from infiltrated DCs and tumor cells as well as other types of cells. However, which type of cells are the primary players to express IFN‐β remains to be determined.

The cGAS is one of the DNA pattern receptors, and can mediate the IFN signaling cascade and trigger strong innate and adaptive immune response.^23^, ^48^ However, activation of the cGAS‐STING signaling is usually impaired in multiple types of cancers due to epigenetic hypermethylation.^28^ This deficiency allows tumor/cancer cells to evade immune surveillance, evidenced by down‐regulated STING expression in TYST cells (data not shown). A previous study reported that cancer‐cell‐intrinsic expression of cGAS, but not STING, promoted the infiltration of effector CD8^+^ T cells and consequently resulted in prolonged survival.^23^ we found that treatment with GPC3_144‐152_ peptide enhanced cGAS expression and γH2AX phosphorylation in the TYST cells co‐cultured with CD8^+^ T cells in vitro. The increased phosphorylated γH2AX can interact with cGAS to activate the cGAS pathway, consistent with previous reports.^28^, ^49^ However, the effect of GPC3_144‐152_ treatment on the cGAS expression and γH2AX phosphorylation was weakened in the cGAS silenced TYST cells. Interestingly, GPC3_144‐152_ treatment almost completely rescued IFN‐β production in the TME, suggesting that IFN‐β may through the JAK2‐STAT3 pathway stimulate a cascade reaction that partially offsets the inhibitory effect of cGAS.^50^ Of cause, how these signaling pathways crosstalk to regulate cytokine production and T‐cell immunity during the progression of TYST remains to be determined.

The activated cGAS‐STING signaling can occur in both tumor cells and immune cells, but it may have unique functions.^45^ The cGAS/STING activation in tumor cells participates in antitumor immune response, while activation of the cGAS/STING pathway in host immune cells mainly contributes to tumor control. On the contrary, we found that treatment with GPC3_144‐152_ peptide in the co‐culture of CD8^+^ T cells with TYST or TYST‐sh‐cGAS cells significantly enhanced the levels of TBK1 and IRF3 phosphorylation in these tumor cells, even after silencing cGAS. The enhanced TBK1 and IRF3 activation should increase the sensitivity of TYST cells to CD8^+^ T‐cell‐mediated killing in the co‐culture system while cGAS silencing usually attenuated the cGAS‐STING signaling to reduce IFN‐β production and CD8^+^ T‐cell infiltration, promoting tumor growth.^51^

This study had limitations, including only one pediatric YST (pYST) cell line. This makes it impossible to accurately assess whether GPC3_144‐152_ peptide can have similar effects in adult YST (aYST). Interestingly, a previous study has shown that there is little difference in molecular signatures and therapeutic responses between primordial germ cell‐originated pYSTs and embryonal carcinoma‐derived aYSTs, suggesting that pYSTs may have responses to antigen‐specific T‐cell immunity, similar to that of aYSTs.^43^ However, we are interested in further comparing them in future studies.

In summary, this study revealed that treatment with GPC3_144‐152_ peptide induced potent antigen‐specific CD8^+^ T‐cell responses in HLA‐A2.1 transgenic mice. Furthermore, treatment with GPC3_144‐152_ peptide, together with adoptive transfer of CD8^+^ T cells, inhibited the growth of TYST tumors, attenuating GPC3 expression and enhancing tumor cell apoptosis in mice. Finally, treatment with GPC3_144‐152_ peptide promoted the infiltration of CD8^+^ T cells into the TME by enhancing the activation of intratumoral cGAS/STING signaling. Our findings may provide a basis for the treatment of TYST, especially for refractory or relapsed TYST, by combination of this therapeutic strategy with other therapies.

## AUTHOR CONTRIBUTIONS

**Junfeng Zhao:** Conceptualization (lead); data curation (lead); formal analysis (lead); investigation (lead); resources (equal); writing – original draft (lead); writing – review and editing (lead). **Le Qin:** Conceptualization (equal); formal analysis (equal); methodology (equal); writing – review and editing (equal). **Guorong He:** Data curation (lead); investigation (equal); methodology (equal); software (equal). **Tiancheng Xie:** Data curation (equal); formal analysis (equal); investigation (equal). **Guanghui Hu:** Data curation (equal); software (equal). **Furan Wang:** Data curation (equal); resources (equal). **Hongji Zhong:** Funding acquisition (equal); supervision (equal); validation (equal). **Jianming Zhu:** Funding acquisition (equal); supervision (equal). **Yunfei Xu:** Conceptualization (equal); funding acquisition (equal); resources (lead); supervision (lead); validation (equal); writing – review and editing (equal).

## CONFLICT OF INTEREST STATEMENT

The authors have no conflict of interest to disclose.

## ETHICS STATEMENT

All animal experiments were conducted according to the protocols of the Institutional Animal Care and Use Committee of Shanghai Tenth People's Hospital and Ningbo Women and Children's Hospital.

## Data Availability

All relevant data are available from the authors upon request.
